# Urban Heat Island Intensity Changes in Guangdong-Hong Kong-Macao Greater Bay Area of China Revealed by Downscaling MODIS LST with Deep Learning

**DOI:** 10.3390/ijerph192417001

**Published:** 2022-12-18

**Authors:** Fan Deng, Ying Yang, Enling Zhao, Nuo Xu, Zhiyuan Li, Peixin Zheng, Yang Han, Jie Gong

**Affiliations:** 1 Key Laboratory of Urban Land Resources Monitoring and Simulation, Ministry of Natural Resources, Shenzhen 518040, China; 2 School of Geosciences, Yangtze University, Wuhan 430100, China; 3 Shenzhen Planning and Natural Resources Data Management Center, Shenzhen 518040, China; 4 Big Data Technology Research Center, Nanhu Laboratory, Jiaxing 314000, China; 5 Institute of Geological Survey, China University of Geosciences, Wuhan 430074, China

**Keywords:** surface temperature, deep residual network, reduced scale, thermal environment change, Guangdong-Hong Kong-Macao Greater Bay Area

## Abstract

The urban heat island (UHI) effect caused by urbanization negatively impacts the ecological environment and human health. It is crucial for urban planning and social development to monitor the urban heat island effect and study its mechanism. Due to spatial and temporal resolution limitations, existing land surface temperature (LST) data obtained from remote sensing data is challenging to meet the long-term fine-scale surface temperature mapping requirement. Given the above situation, this paper introduced the ResNet-based surface temperature downscaling method to make up for the data deficiency and applied it to the study of thermal environment change in the Guangdong-Hong Kong-Macao Greater Bay Area (GBA) from 2000 to 2020. The results showed (1) the ResNet-based surface temperature downscaling method achieves high accuracy (R^2^ above 0.85) and is suitable for generating 30 m-resolution surface temperature data from 1 km data; (2) the area of severe heat islands in the GBA continued to increase, increasing by 7.13 times within 20 years; and (3) except for Hong Kong and Macau, the heat island intensity of most cities showed an apparent upward trend, especially the cities with rapid urban expansion such as Guangzhou, Zhongshan, and Foshan. In general, the evolution of the heat island in the GBA diverges from the central urban area to the surrounding areas, with a phenomenon of local aggregation and the area of the intense heat island in the Guangzhou-Foshan metropolitan area is the largest. This study can enrich the downscaling research methods of surface temperature products in complex areas with surface heterogeneity and provide a reference for urban spatial planning in the GBA.

## 1. Introduction

Urban thermal environment refers to the performance state of the thermal field in an urban space environment, which is influenced by the physical properties of the underlying surface and the social and economic activities of human beings [1,2]. With the urbanization process in China, the urban built-up area has expanded significantly, while the vegetation coverage and water area have decreased. As a result, the urban thermal environment deteriorates, the urban heat island effect becomes more and more serious, and the livable degree decreases. The first task of mitigating the urban thermal environment is to accurately monitor the changes in the urban thermal environment so that we can take timely and proper prevention and control measures, to reduce the threat of thermal environment deterioration.

The Guangdong-Hong Kong-Macao Greater Bay Area (GBA) is one of the fastest urbanizing regions in China and one of the most populous globally, with an urbanization rate of over 80 percent. This region’s urban system and spatial structure have undergone fundamental changes in recent decades [3]. In the urbanization process in this region, independent heat island areas (high-temperature areas) are gradually linked and interact with each other, resulting in regional heat islands and regional thermal environment problems. To a certain extent, it intensifies urban air pollution and leads to the increase in extreme weather events and local secondary disasters; it significantly increases the consumption of urban production and domestic energy, exacerbates global warming; it increases the incidence of heat-related diseases such as heart disease and cardiopulmonary disease in urban residents [4]. Understanding the impact of rapid urbanization on the dynamics and evolution of the regional surface thermal environment can provide policy guidance for the GBA and scientific reference for the environmental governance of urban agglomeration under rapid growth urbanization.

Surface temperature is the essential parameter for monitoring the thermal environment. However, urban surface temperature (LST) has high temporal and spatial heterogeneity, which is challenging to meet the needs of ground observation [5,6]. Remote sensing is essential for obtaining high spatiotemporal heterogeneity of surface temperature data. Urban thermal environment research usually needs urban surface temperature data with a high temporal and spatial resolution to meet the requirements. Therefore, it is an essential method of remote sensing thermal environment monitoring to convert the urban surface temperature data with a high temporal and low spatial resolution to obtain the urban surface temperature products with a fine spatio-temporal resolution that meets the application requirements [7,8].

LST obtained by satellites currently restricts each other in terms of spatial and temporal resolution. Such as MODIS surface temperature product with high temporal resolution (revisit period of 1 day) but a low spatial resolution (1 km) and Landsat 8 TIRS with a high spatial resolution (100 m) but a long revisit period (16 days) [9]. In addition, the sensors of existing high spatial resolution satellites usually do not have a thermal infrared band, which leads to the contradiction between surface temperature data’s temporal and spatial resolution [10]. When monitoring an urban heat island, a very low spatial resolution will lead to the inability to capture the spatial details of ground objects, and a very low temporal resolution will easily reduce the practicability of acquiring satellite images due to cloud interference, so it is impossible to obtain the change characteristics of time series of surface temperature.

The mainstream methods to improve the spatiotemporal resolution of surface temperature include the spatio-temporal fusion and downscaling [8,9]. The first method requires fewer auxiliary parameters, but the accuracy uncertainty increases when the period between the reference time and prediction time is large (the land cover type changes greatly). The second method is based on the invariance of scale and introduces the concept of “downscaling”. By establishing the regression relationship between coarse-resolution surface temperature and other surface parameters, then applying this relationship to high-resolution surface parameters, the high-resolution surface temperature can be predicted [10,11]. This method can directly downscale and decompose low-resolution images, which has certain advantages in high heterogeneity areas, but the rationality of environmental covariate selection will greatly affect the prediction accuracy [11].

Machine learning methods have also been used to study LST downscaling in recent years. For example, Gao et al. developed the data mining sharpener (DMS) algorithm, which uses decision tree regression to downscale Landsat land surface temperature, generates 30 m long-time series data, and monitors the Washington agricultural area of the United States [12]. Usually, the downscaling of surface temperature needs other surface parameters, such as various band indexes (NDVI, NDBI, SAVI, etc.) and elevation information. Most algorithms use fewer or single indexes, and the accuracy is low in areas with high heterogeneity. For example, using a single vegetation index (such as NDVI) has high simulation accuracy in densely vegetated areas, but the error is significant in building areas. Downscaling based on deep learning considers multiple indexes in feature selection and identifies their internal statistical patterns, which can realize the regression analysis of multiple environmental covariates [13].

Therefore, this study aims to build a deep neural network to establish the relationship between coarse resolution surface temperature and surface type. Assuming that this relationship does not change with the improvement of resolution, then input the high-resolution surface type variables into the model to obtain the 30-m resolution surface temperature of 11 cities in Guangdong, Hong Kong, and Macao Special Administrative Regions (SAR), including Hong Kong, Macao, Guangzhou, Shenzhen, Zhuhai, Foshan, Zhaoqing, Huizhou, Dongguan, Zhongshan, and Jiangmen. Then, based on the long-time series and high spatial-temporal resolution surface temperature after downscaling, the intensity of heat islands in Guangdong, Hong Kong, and Macao Bay area is characterized and leveled, and its interannual evolution law is analyzed. Finally, it provided theoretical support for feasible suggestions for GBA to promote rational and orderly urban expansion, alleviate the urban heat island effect and guide the city’s sustainable development.

## 2. Study Area and Data

### 2.1. Study Area

**Figure 1:**
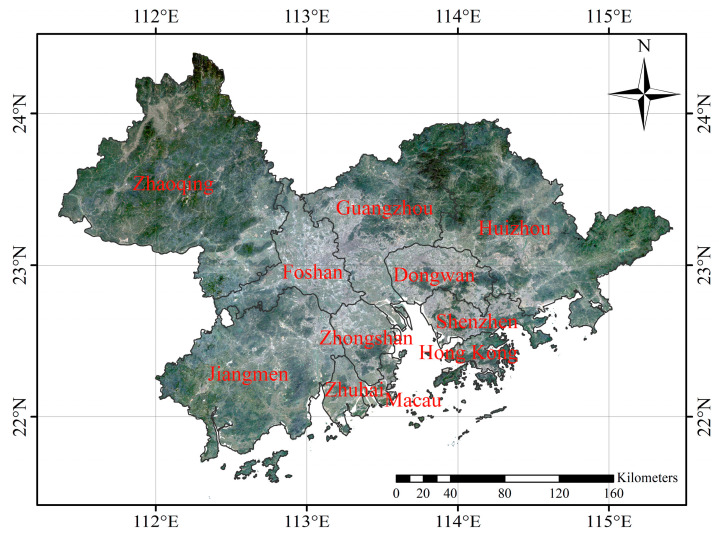
The location of Guangdong-Hong Kong-Macao Greater Bay Area.

Guangdong-Hong Kong-Macao Greater Bay Area (GBA) is located between 21.57° N~24.39° N and 111.36° E~115.41° E, with a total area of 5.5 × 10^4^ km^2^, composed of Hong Kong and Macao SAR and Guangzhou, Shenzhen, Zhuhai, Foshan, Huizhou, Dongguan, Zhongshan, Jiangmen, and Zhaoqing in Guangdong Province (Figure 1). It is one of the regions with the highest degree of openness and the most economic vitality in China. GBA is a compound Delta, which is formed by the accumulation of sediment brought by the lower reaches of Xijiang, Beijiang, and Dongjiang rivers of the Pearl River System in the estuary. It is located in the south subtropical zone and belongs to the subtropical marine monsoon climate. It has abundant rainfall, sufficient heat, and the same period of rain and heat. The annual average temperature is 21.9 °C. Affected by the monsoon climate, the rainfall is concentrated from April to September, and the annual average rainfall is 1600~2300 mm. Northerly wind prevails in winter and the weather is dry; Southwest and Southeast winds prevail in summer, with high temperature and rainy weather.

### 2.2. Data

The data used in this study include MOD11A2, MCD12Q1, Landsat 5 TM, Landsat 8 OLI, DEM elevation data, and administrative region vector data. The description of each data is shown in Table 1. These surface temperature products and Landsat data were acquired during the daytime from June 1 to August 31. Remote sensing products and data are acquired and processed on Google Earth Engine (GEE).

Using the mono-window algorithm to calculate the LST as a feature layer, we choose the calibrated top-of-atmosphere (TOA) reflectance product of Landsat, which contains two resampled thermal infrared bands with a 30 m resolution. Current practice for LST retrieval involves the use of remote sensing images from sources like MODIS or Landsat, along with mono-window, split-window, or atmospheric correction algorithms. (Cristóbal et al., 2018) [14]. The mono-window algorithm is an LST retrieval algorithm proposed by Qin, Karnieli, and Berliner (2001) [15] in response to the presence of just one thermal infrared band for (TM) data (Wang et al., 2015) [16], and is used to retrieve LST in this study as:

(1) $$\mathrm{Ts}=\left\{a\left(1-C-D\right)+\left[b\left(1-C-D\right)+C+D\right]T_{b}-{\mathrm{DT}}_{a}\right\}/C$$

where Ts represents the retrieval LST; $T_{a}$ represents the atmospheric mean acting temperature; $T_{b}$ represents the brightness temperature; a and b are −67.355351 and 0.458606, respectively; C and D are obtained by Equations (2) and (3):

(2) C=ε∗τ

(3) D=(1−τ)[1+(1−ε)τ]

where τ is the atmospheric transmissivity. τ and $T_{a}$ were calculated based on atmospheric parameters derived from NASA’s website (https://atmcorr.gsfc.nasa.gov/, accessed on 20 April 2022). ε represents surface emissivity based on NDVI and vegetation coverage (Equations (4) and (5)):

(4) $$\varepsilon_{s}=0.9625++0.0614F_{v}-0.0461F_{v^{2}}$$

(5) $$\varepsilon_{b}=0.9589+0.086F_{v^{2}}-0.061F_{v^{2}}$$

In Equations (4) and (5), $\varepsilon_{s}$ and $\varepsilon_{b}$ are the emissivity of natural surface and urban, respectively. $F_{v}$ is vegetation coverage.

The vector data was downloaded from China National Catalogue Service for Geographic Information (https://www.webmap.cn/main.do?method=index, accessed on 20 April 2022).

## 3. Method

**Figure 2:**
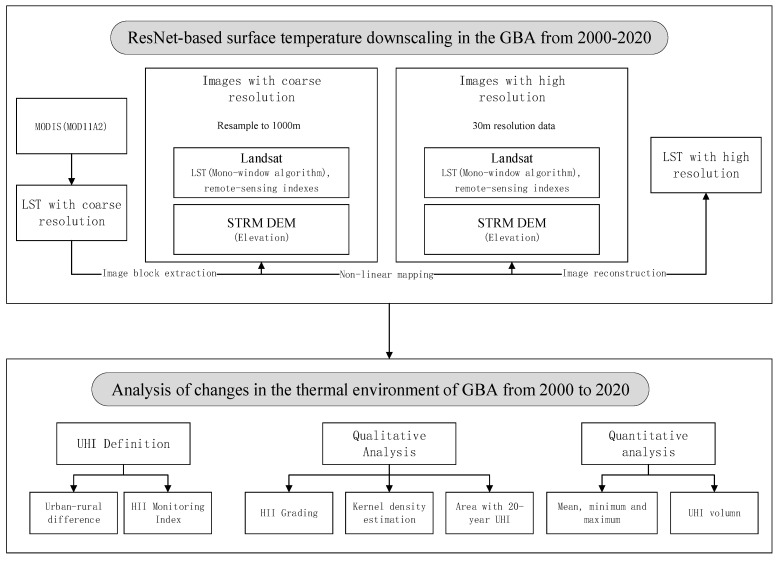
Technical flow chart.

Due to the low temporal resolution of Landsat and the high frequency of cloud coverage in the study area, it is difficult to obtain remote sensing images with good spatio-temporal consistency. One goal of the study was to downscale the MODIS 1000 m surface temperature product to 30 m resolution of the surface temperature data. First, remote sensing indices such as NDVI, SAVI, MNDWI, NDBI, NDISI, and BI were calculated from Landsat data, and the land surface temperature was retrieved based on Landsat. Next, the relationship between these indicators and MODIS surface temperature products was extracted by applying the ResNet model, and these relationships were applied to Landsat data to generate high-resolution surface temperature data. These data were used to analyze the changes in the thermal environment in GBA from 2000 to 2020. The specific technical process is shown in Figure 2.

### 3.1. Downscaling Method

Land surface temperature downscaling is a method to retrieve land surface temperature data with higher spatial resolution than the original data based on independent variables. Independent variables represent the biophysical characteristics obtained from high spatial resolution remote sensing data [17]. This method uses the relationship between the surface temperature and the image pixels with coarse spatial resolution but a short revisit period and assumes that the relationship does not change with the improvement of spatial resolution, so as to obtain the surface temperature data with high spatial resolution and short revisit time according to the image with long revisit period but high spatial resolution.

Traditional downscaling methods are most successfully used in areas with uniform terrain and the single land cover type, but their effectiveness is rarely verified in areas with complex terrain and complex land cover [18,19]. Therefore, other relevant variables have been applied in many studies rather than predictors based on separate remote sensing indexes [19,20,21,22,23]. Bechtel et al. [21] introduced additional parameters, such as average TIR images, land cover products, and data obtained from image dimensionality reduction (such as principal component analysis), which improved the goodness of fit in the downscaling model.

Many scholars believe that the application of simple multivariate modeling can improve the accuracy of surface temperature downscaling. The results obtained by Maeda et al. [7] show that when DEM is combined with NDVI, there will be a high determination coefficient (R^2^ ≥ 0.95). In addition, Duan and Li et al. [22] proposed using geographically weighted regression (GWR) and taking NDVI and digital elevation model (DEM) as variables to downscale MODIS surface temperature products, which can reduce the stationary effect affected by simple univariate and multiple regression methods.

Traditional statistical techniques have achieved some results in many case studies, but some scholars suggest using machine learning technology to consider the nonlinear relationship between prediction variables and surface temperature [17,20]. Machine learning approaches can incorporate more explanatory variables into deterministic physical modeling even with mathematically more complex regression models. Different techniques have been developed for downscaling research, including Bayesian-based modeling [24], support vector regression [25], artificial neural networks [26] and random forests [13,18]. For the first time, Hutengs and Vohland et al. [27] used random forest techniques for downscaling, and they downscaled Landsat thermal infrared images with spatial resolutions of 60 m and 120 m to 30 m, which is consistent with the spatial resolution of other bands of Landsat. They fused multi-source data in the model and verified the good performance of the machine learning model. The ability of machine learning algorithms to deal with nonlinear relationships has a great advantage in downscaling surface temperatures. As a new generation of machine learning methods, deep learning is very promising in many research fields [28,29,30]. Therefore, it is meaningful to study whether deep learning techniques show more advantages in reducing the surface temperature for complex nonlinear problems.

This study will use ResNet (Residual Networks) for surface temperature downscaling. ResNet is a widely used CNN feature extraction network [31]. The main idea is to add a channel of connection between the input channel and the output channel, called skip connection, so that the information can be output directly from the input, and solve the vanishing/exploding gradient problem. At the same time, it is not necessary to learn the whole process when training the network, only the output residual of the previous part needs to be learned. It not only saves time but also reduces the learning difficulty of the neural network.

After analysis, we selected 8 features with good discrimination to simulate the relationship between surface temperature and surface types from 2000 to 2020 (Table 2, Equation (6)). The eight features are DEM, surface temperature (LSTm, calculated using a single-window algorithm), normalized vegetation index (NDVI), soil-adjusted vegetation index (SAVI), improved normalized water body index (MNDWI), and normalized building index (NDBI), normalized impervious surface index (NDISI) and bare ground index (BI). In previous studies, these features have been shown to have a strong relationship with surface temperature [32,33].

(6) $${\mathrm{LSD}}_{D}=f\left(\mathrm{DEM},\;\mathrm{NDVI},\mathrm{SAVI},\mathrm{MNDWI},\mathrm{NDBI},\mathrm{NDISI},\mathrm{BI},{\mathrm{LST}}_{m}\right)$$

f( ) refers to the nonlinear function that the ResNet model needs to model.

First, resample these features to 1000-m resolution as the independent variable of the downscaling model, and obtain the relationship between the surface temperature and each feature at 1000-m resolution.

Then, the dependent variable is the surface temperature value of the MODIS surface temperature product and considering that satellite data may be vacant or anomalous due to cloud cover or pollution and sensor failures [40,41,42], unpopulated and anomalous data need to be eliminated to establish valid data pairs.

In the regression process, 19,000 samples are used as the training data set, and 2700 samples are used as the test data set. In order to reduce overfitting and obtain more reliable classification results, K-Cross Validation (K-CV) analysis method is used to determine the ratio of the training set and test set.

#### 3.1.1. Model Structure

In this paper, the ResNet-50 model is used to downscale the surface temperature. The ResNet-50 network is a residual convolutional neural network with a structure of 50 layers, which consists of three parts: independent convolution structure, residual convolution structure, average pooling, and fully connected structure. The independent convolution structure uses a convolution kernel with a size of 7 × 7 and a channel number of 64 to convolve the image and a 3 × 3 convolution kernel for max-pooling operation. The residual convolutional structure consists of 4 different convolutional residual modules, and the residual branch of each convolutional residual module consists of two convolutions, batch regularization, and ReLu. Taking the residual module as the unit, the residual module operations of 3, 4, 6, and 3 times are performed for each residual convolution structure. Finally, flatten the feature vector, perform average pooling and full connection processing on the network, and output the segmentation map, thereby effectively avoiding the problem of network degradation caused by the disappearance of the network gradient. The model structure is shown in Table 3, and the hyperparameter settings are shown in Table 4. The model optimizer uses Adam (Adaptive Moment Estimation), which has many advantages, such as less memory, high computational efficiency, and is suitable for non-steady-state targets. The method uses the system’s first-order moment estimation and second-order moment estimation to dynamically adjust each parameter’s learning rate gradient.

#### 3.1.2. Model Validation

To evaluate the model’s performance, a ten-fold cross-validation approach was employed to test the model’s predictive ability and the overfitting problem [42]. In cross-validation, all samples are randomly divided into ten groups of roughly equal size. Each group was sequentially kept as a validation dataset to evaluate model performance, and the remaining 9 groups were used for model fitting. This process is repeated ten times, and each group is used to validate the model once as part of the validation.

Each sample on the MODIS surface temperature product (resolution 1000 m) corresponds to about 1111 pixels on the downscaled surface temperature (resolution 30 m), and the average of these 1111 pixels is used to fit the MODIS sample.

Through the statistical indicators Pearson correlation coefficient (R^2^) and root mean square error (RMSE), the estimated results are compared with the observation results of actual meteorological observation stations to evaluate the accuracy of the model. The downscaling model is established using the data of the training set, and then the accuracy of the established model is evaluated using the data of the test set.

(7) $$R^{2}=1-\frac{\sum \left({\mathrm{LST}}_{O}-{\mathrm{LST}}_{D}\right){)}^{2}}{\sum \left({\mathrm{LST}}_{O}-\bar{{\mathrm{LST}}_{D}}\right){)}^{2}}$$

(8) $$\mathrm{RMSE}=\sqrt{\frac{1}{n}\sum_{i=1}^{n}\left({\mathrm{LST}}_{O}-{\mathrm{LST}}_{D}\right){)}^{2}}$$

where n is the number of pixels participating in the evaluation, ${\mathrm{LST}}_{O}$ is the surface temperature of the original image, ${\mathrm{LST}}_{D}$ is the surface temperature of the downscaled image, and $\bar{{\mathrm{LST}}_{D}}$ is the average of the surface temperature in the entire downscaled image.

### 3.2. Analysis Method of Thermal Environment Change

#### 3.2.1. Urban-Rural Difference Method

According to the definition of an urban heat island, its effect is characterized as the phenomenon that cities are warmer than suburban or rural areas. This relative warming effect is usually expressed as the difference between the urban surface temperature and the average suburban temperature. In this paper, Heat Island Intensity (HII) is defined as the difference between the surface temperature of each pixel in the urban area and the average surface temperature of the rural area in the image.

(9) $${\mathrm{HII}}_{\mathrm{ij}}=T_{\mathrm{ij}}-\bar{T_{r}}$$

In Equation (9), ${\mathrm{HII}}_{\mathrm{ij}}$ and $T_{\mathrm{ij}}$ are the heat island intensity value and the surface temperature value of the pixel at the spatial position i and j in the urban area, respectively; $\bar{T_{r}}$ is the average surface temperature in a rural area.

#### 3.2.2. Heat Island Intensity Monitoring Index

The Heat Island Intensity Monitoring Index (HIMI) [43] was used to represent the study area’s dynamic characteristics of the heat island effect. Firstly, the heat island levels are classified according to the range of heat island intensity in the study area. The classification criteria are shown in Table 5. Then, the heat island intensity monitoring index is calculated according to Equation (10).

(10) $${\mathrm{HIMI}}_{i}=G_{i,2020}-G_{i,2000}$$

In the formula: G_i,2000_ and G_i,2020_ are the heat island levels of the pixels in the study area in 2000 and 2020, respectively. For example, HIMI_i_ > 0 means that the corresponding pixel i has changed from the low heat island level in 2000 to the high heat island level in 2020, which is defined as a heat island deterioration area; HIMI_i_ = 0 means that the heat island level of the corresponding pixel i remains unchanged, and is defined as a heat island Stability area; similarly, HIMI_i_ < 0, defined as heat island mitigation area.

## 4. Results and Analysis

### 4.1. Surface Temperature Downscaling

**Figure 3:**
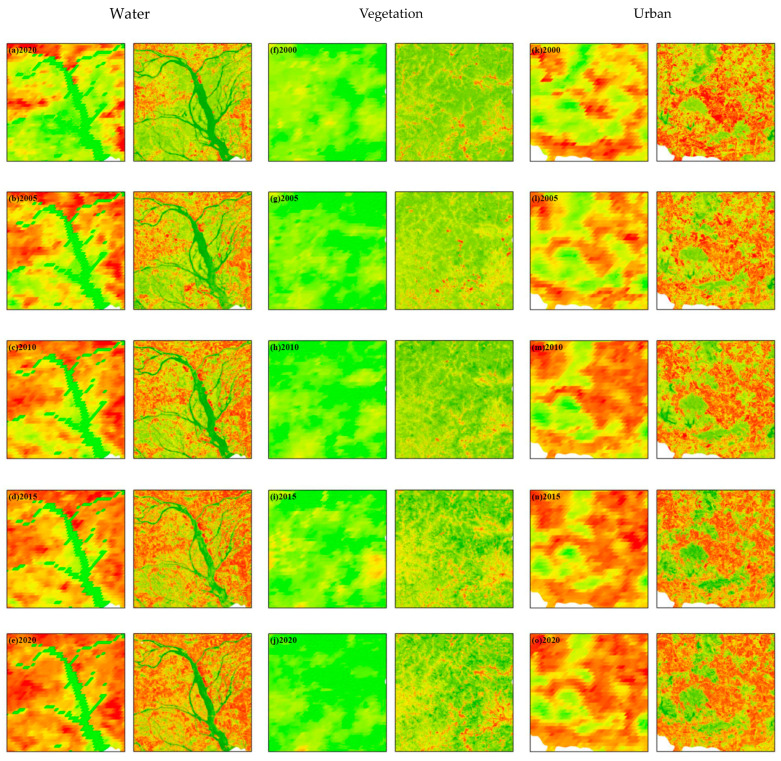
MODIS LST downscaling effect of different land cover regions (water, vegetation, and urban).

Three land cover types, namely high vegetation coverage area, water body, and urban construction land, are selected to evaluate the downscaling effect (Figure 3).

For the river region, in the coarse resolution image of 2000, the high-temperature region is located in the upper, left middle and right edge of the image. The downscaled high-resolution LST images show a consistent distribution, but with more detail due to the narrow width of the river. Some small rivers are hard to present in low-resolution images. In other years, coarse-resolution and high-resolution data also show the same distribution characteristics. In rural areas, large areas of land are covered by vegetation, and there is a lack of detail in low-resolution LST data. As a result, some subtle surface heterogeneity is difficult to be visualized. The boundary of MODIS surface temperature objects at a spatial resolution of 1000 m is blurred and cannot show the details of temperature distribution in some areas. Surface temperature images downscaled to 30 m reveal more spatial detail. The spatial heterogeneity of surface temperature is more obvious.

It was found that the original 1000 m surface temperature wass consistent with the surface temperature information features in the downscaling results, and the high-temperature and low-temperature regions are highly consistent, indicating that the downscaling results preserve the distribution of thermal features of the original surface temperature product images.

**Figure 4:**
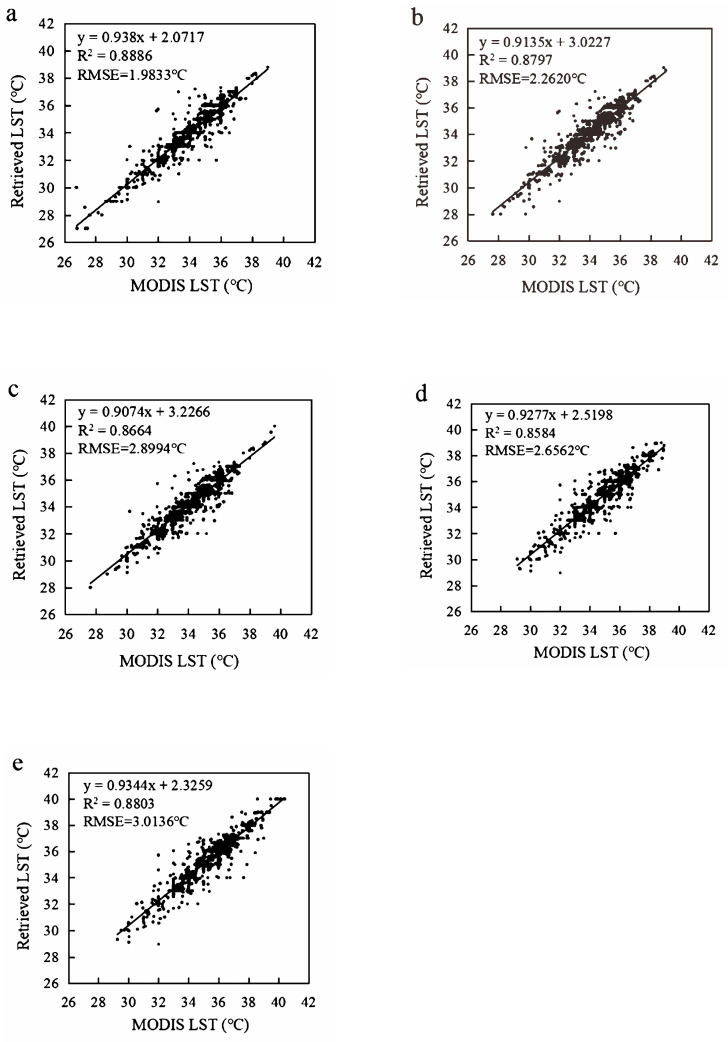
MODIS LST downscaling precision evaluation. ((**a**–**e**): 2000, 2005, 2010, 2015, 2020).

The results of MODIS surface temperature downscaling in the Greater Bay Area were validated using Pearson’s correlation coefficient (R^2^) and root mean square error (RMSE). In the five periods selected in this study, R^2^ was above 0.85 and RMSE was between 1.9833 °C and 3.0136 °C (Figure 4), indicating good accuracy of downscaling results. This shows that the temperature downscaling method is suitable for this study area.

### 4.2. Thermal Environment Changes in the Greater Bay Area

In this study, the ResNet downscaling model was used to obtain the land surface temperature of the Greater Bay Area in 2000, 2005, 2010, 2015, and 2020 with a resolution of 30 m, and the rural-urban difference method and heat island intensity monitoring index were used to classify the heat island intensity levels in the GBA. The temporal and spatial dynamic changes of heat islands in five periods in the Greater Bay Area were analyzed.

#### 4.2.1. Heat Island Expansion

**Figure 5:**
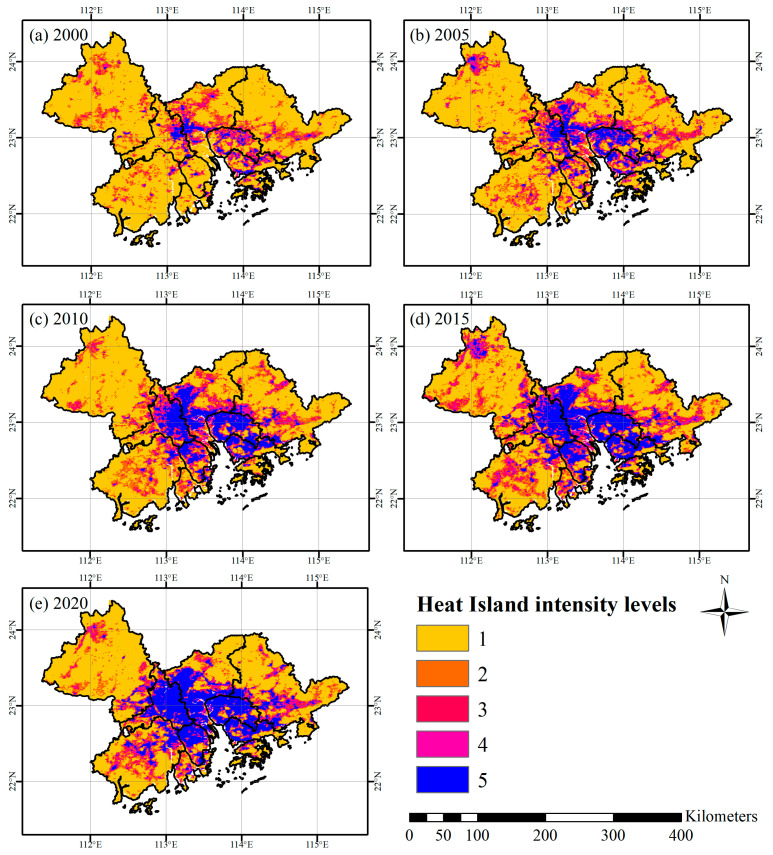
GBA heat Island intensity levels distribution from 2000~2020.

According to the classification of heat island intensity levels in Table 5, the main areas of the distribution of the five levels of heat island intensity are shown in Figure 5. The region of the UHI shows a very significant expansion, especially the strong UHI region (blue region). It forms a spatially continuous large intense heat island region across the administrative area. In 2000, the high-intensity region was mainly distributed in the west of Guangzhou and Shenzhen, the east of Foshan and Jiangmen, the middle of Dongguan, and the coastal areas of the Hong Kong Special Administrative Region. By 2020, except for Zhaoqing, other cities in the Greater Bay Area have obvious high heat island intensity areas and form a concentrated and contiguous distribution in the central and southern regions.

In addition, we calculated and counted the changes in the area of the five levels of the heat island at five-time points (Table 6). Most notably, the fifth level of the HII region has experienced a dramatic increase. By 2020, its area had increased to 7.13 times that of 2000. Generally, areas with high heat island intensity showed dramatic growth, and the areas with high-intensity heat islands (levels 4 and 5) increased by 170.1%. Accordingly, the area of the low-intensity UHI (levels 1 and 2) decreased by a total of 24.8%.

**Figure 6:**
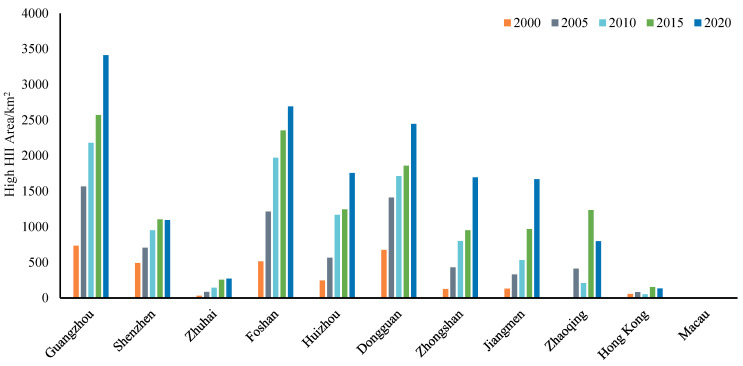
The changes in the high heat island area in eleven cities of GBA from 2000~2020.

According to the above HII levels, the area covered by the strong heat island area (HII > 4 °C) was calculated. Since the heat island distribution of each city in the GBA has its special characteristics of expansion, the intensity, and area of the high HII area of 11 cities in the GBA are statistically analyzed, and the results are shown below (Figure 6). It can be seen that Guangzhou has the largest area of high HII area and the most drastic increase in its area, followed by Foshan and Dongguan, and Macao has the smallest area of high HII area and the smallest change, followed by Hong Kong. Shenzhen, as a sub-provincial city in Guangdong Province and one of the central cities in the GBA, does not have a prominent high HII area, and the magnitude of change is relatively flat.

The differences in changes in urban heat islands in various cities also reflect the development process and urbanization of these cities in the Greater Bay Area. Macau and Hong Kong are developed-cities, with high levels of urbanization and strict ecological and environmental protection. Therefore, the urban heat island effect is relatively stable. The Shenzhen Special Economic Zone has a relatively small area; its development started earlier and matured. Therefore, the urban heat island effect has not changed much in the past five years. Furthermore, Zhuhai has a similar situation to Shenzhen.

#### 4.2.2. Analysis of Interannual Heat Island Intensity Variation

**Figure 7:**
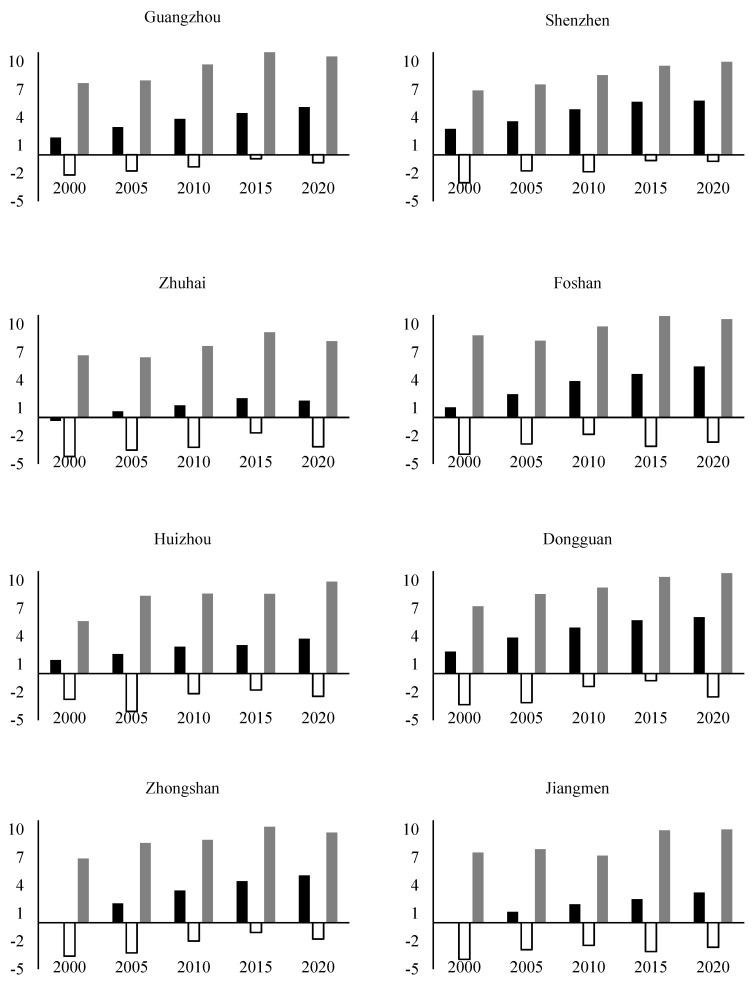

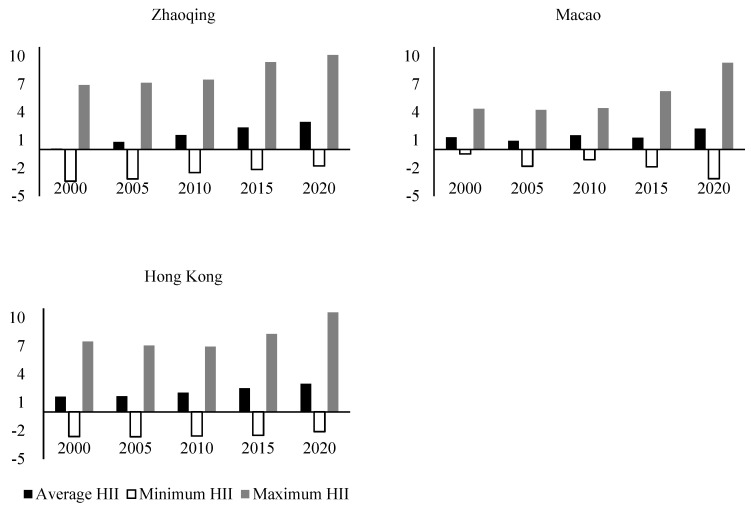
The changes in the average, minimum and maximum value in eleven cities of GBA from 2000~2020.

The maximum, minimum, and average values of the heat island intensity in each city over the past 20 years were calculated, and different cities present different situations (Figure 7). The black bar graph shows the average change of the interannual heat island intensity, and the value is between 0.04 °C and 6.06 °C.

Most of the Mainland Chinese cities, such as Zhongshan, Foshan, Dongguan, Shenzhen, and Guangzhou, showed a rapid increase trend in heat island intensity. The city with the fastest increase in average heat island intensity was Zhongshan, with an average heat island intensity increase of 1.24 degrees Celsius every five years. In developed cities, such as Hong Kong and Macau, the average heat island intensity is more stable or growing more slowly.

Notably, some smaller cities, such as Zhongshan, Jiangmen, and Zhaoqing, did not have or showed a mild heat island effect in 2000, and then began to grow rapidly. On the contrary, the heat island intensity of other cities showed a stable or even decreased phenomenon from 2015 to 2020. Such as Hong Kong, Macau, Zhuhai, Shenzhen, and other cities. This situation is because the time of modernization development between cities is inconsistent. As developed cities, Hong Kong and Macao developed earliest. Guangzhou, the capital of Guangdong Province, and Zhuhai, Shenzhen, China’s earliest special economic zones have been developed since the late 1970s. The radiation of surrounding cities drives the rapid development of Zhongshan, Jiangmen, Zhaoqing, and other small cities.

#### 4.2.3. Heat Island Intensity Monitoring Index Changes

**Figure 8:**
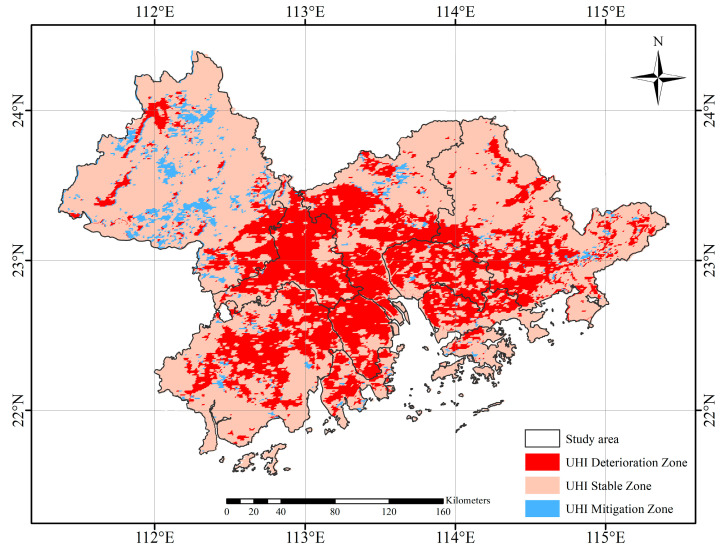
GBA heat island intensity monitoring index changes from 2000~2020.

Based on the Heat Island Intensity Monitoring Index, areas are defined as heat-to-exacerbation areas, heat island stabilization areas, and heat island mitigation areas from 2000 to 2020 (Figure 8). The heat island deterioration areas are concentrated and contiguous, mainly in the southwest of Guangzhou, the north of Shenzhen, the southwest of Huizhou, the middle and east of Jiangmen, the north of Hong Kong SAR, Foshan, Dongguan, Zhongshan, and Zhuhai. The area of the heat island deterioration area is approximately 30,470.2 km^2^. The heat island stabilization zones are distributed in the north of Zhaoqing and Guangzhou, the northeast of Huizhou, and the southwest of Jiangmen, with an area of about 31,604.5 km^2^. The heat island mitigation areas are scattered in Guangzhou, Zhaoqing, Huizhou, Jiangmen, and Hong Kong SAR, with an area of about 19,918.2 km^2^. The area of the heat island deterioration area is similar to that of the heat island stabilization area, while the area of the heat island mitigation area is much smaller than that of the deterioration area.

## 5. Discussion

Studying the distribution and changes of the heat island in the urban agglomeration of the GBA and its relationship with urban development has important practical significance for the planning and improvement of the ecological environment of the GBA and even for alleviating the thermal environment problems of other urban agglomerations.

Thermal environmental monitoring often requires surface temperature data with high spatial resolution and short revisit periods to meet research needs. Since the spatial resolution and revisit period of existing surface temperature product data cannot meet the application requirements of this study, a surface temperature downscaling method based on a deep residual network (ResNet) is proposed. The MODIS LST product with a 1000-m spatial resolution was downscaled to 30 m using the ResNet based on the relationship between LST and elevation, NDVI, SAVI, MNDWI, NDBI, MNDISI, BI, and LST_m_. The model achieved high precision with an R^2^ of about 0.85 and RMSE of 1.9833~3.0136 °C, which is higher than our previous study using random forest [44]. Some other nonlinear models were used to downscale LST and achieve higher precision [45,46], such as nonlinear, regressive models and multi-factor, geographically weighted machine learning algorithms. Compared with these methods, the downscaling method based on deep learning is more suitable for large-scale data processing, and it avoids the selection process of surface feature parameters, which has an important impact on the accuracy of the above methods.

This study did not specifically use land cover type as a parameter when retrieving LST. However, the LST inversion method we used takes into account the differences in the emissivity of different land cover types by simulating the natural and urban surface emissivity by vegetation cover. The LST downscaling accuracy is determined by many remote sensing indices and other parameters in this study, and the deep learning method can learn the patterns through a large number of samples, so the inversion accuracy will not be significantly reduced by a certain parameter. A more accurate simulation of the emissivity differences of different land cover types is conducive to a more accurate inversion of surface temperature, which is very beneficial to the study of small-scale thermal environments [47,48].

The spatial distribution map of the heat island effect with 30 m resolution in summer was obtained. The data included fine-scale characterization and change monitoring of the heat island effect in the GBA’s five periods (2000~2020). With cities’ rapid expansion, the GBA’s urban heat island has expanded significantly, and the heat intensity has continued to increase. The area of high-intensity heat islands in 2020 is more than seven times larger than that in 2000. Except for Hong Kong and Macau, the heat island intensity of most cities showed an obvious upward trend, especially the cities with rapid development, such as Guangzhou, Zhongshan, and Foshan. In short, the urban heat island area in the GBA urban agglomeration has crossed the city boundary, forming a continuous and huge heat island area. Due to the differences in the degree of urban development, urban heat island changes in different cities also show different characteristics, which is reflected in the fact that the heat island intensity of Zhongshan and Foshan has increased rapidly, while that of Hong Kong and Macao has the slowest growth rate.

Rapid urbanization coupled with the climate background of global warming, urban residents in the GBA region are gradually exposed to increasing urban high temperatures and extreme heat wave events. Through the observation and analysis of meteorological stations in 183 cities in China for 26 years, GBA is the area where solar radiation increases the most, and it is also the area where the PET (Physiological equivalent temperature) increases most rapidly [49]. GBA also is the area with the most serious loss of urban green space. Furthermore, measures such as adjusting urban structure, increasing vegetation area, and changing building colors are used to alleviate the heat island effect in areas with persistent high calorific values, and create a cool buffer zone between people and high-temperature areas to help residents cope with heat island effect [50,51]. In rapidly developing and highly urbanized areas, it is necessary to scientifically determine the scope of urban development in urban planning and design the minimum ecological safety distance, prevent further deterioration of the surface thermal environment in the new urban areas of the GBA, and protect low-temperature areas.

## 6. Conclusions

In this study, the surface temperature downscaling method used was highly accurate, with an R^2^ value above 0.85. The analysis of urban heat island intensity in the Guangdong-Hong Kong-Macao Greater Bay Area from 2000 to 2020 based on this downscaled data shows that the heat island area in the region has significantly expanded, with a significant increase in heat island intensity in most cities, particularly in developing cities. In summary, the proposed downscaling method has a certain reference value for the land surface temperature product downscaling in the area with complex surface heterogeneity, and the findings support regional thermal environment improvement and urban agglomeration spatial planning.

## Figures and Tables

**Table 1:** Description of data used in this study.

Data Set	Data Acquisition Date	Spatial Resolution	Purpose
MOD11A2	1 June 2000–31 August 2000 1 June 2005–31 August 2005 1 June 2010–31 August 2010 1 June 2015–31 August 2015 1 June 2020–31 August 2020	1 km	Downscaling
MCD12Q1	2001, 2005, 2010, 2015, 2020	500 m	Extract urban area
Landsat 5 TM	1 June 2000–31 August 2000 1 June 2005–31 August 2005 1 June 2010–31 August 2010 1 June 2015–31 August 2015 1 June 2020–31 August 2020	30 m	Calculate the remote sensing index and provide downscale variables
Landsat 8 OLI	1 June 2000–31 August 2000 1 June 2005–31 August 2005 1 June 2010–31 August 2010 1 June 2015–31 August 2015 1 June 2020–31 August 2020	30 m	Calculate the remote sensing index and provide downscale variables
SRTM DEM (V3)	2000	30 m	Obtain the elevation value of the study area
Vector data	2017	1:1,000,000 1:250,000	Administrative map

**Table 2:** Description of spectral indices used in this study.

Remote Sensing Index	Formula	Description
NDVI	NDVI = (NIR − Red)/(NIR + Red)	The normalized vegetation index can characterize vegetation growth and vegetation coverage. [34]
SAVI	SAVI = [(NIR − Red)(1 + L)]/(NIR + Red + L) (L is the soil regulating factor)	Eliminate the interference of soil background on vegetation index. [35]
MNDWI	MNDVI = (Green − MIR_1_)/(Green + MIR_1_)	Used to highlight water body information in imagery. [36]
NDBI	NDBI = (MIR − NIR)/(MIR + NIR)	Using the law that the reflectivity of building land in the mid-infrared band is higher than that in the near-infrared band, this index is helpful for the extraction of building land. [37]
NDISI	$\mathrm{NDISI}=\frac{\mathrm{TIR}-\left[\left(\mathrm{MNDWI}+\mathrm{NIR}+{\mathrm{MIR}}_{1}\right)/3\right]}{\mathrm{TIR}+\left[\left(\mathrm{MNDWI}+\mathrm{NIR}+{\mathrm{MIR}}_{1}\right)/3\right]}$	The index can effectively distinguish impervious surface and soil and suppress the influence of sand and water information. [38]
BI	$\mathrm{BI}=\frac{\left({\mathrm{MIR}}_{2}+\mathrm{Red}\right)-\left(\mathrm{NIR}+\mathrm{Green}\right)}{\left({\mathrm{MIR}}_{2}+\mathrm{Red}\right)+\left(\mathrm{NIR}+\mathrm{Green}\right)}$	Bare soil can be distinguished from other features by this index. [39]

**Table 3:** ResNet-50 network structure.

Layer Name	Output Size	ResNet-50
Input	96 × 96	-
Conv	48 × 48	7 × 7, 64, stride 2
		3 × 3 max pool, stride 2
Conv2_x	24 × 24	$[\begin{array}{c} 1\times 1\\ 3\times 3\\ 1\times 1 \end{array}\begin{array}{c} 64\\ 64\\ 256 \end{array}]\times 3$
Conv3_x	12 × 12	$[\begin{array}{c} 1\times 1\\ 3\times 3\\ 1\times 1 \end{array}\begin{array}{c} 64\\ 64\\ 256 \end{array}]\times 4$
Conv4_x	6 × 6	$[\begin{array}{c} 1\times 1\\ 3\times 3\\ 1\times 1 \end{array}\begin{array}{c} 64\\ 64\\ 256 \end{array}]\times 6$
Conv5_x	3 × 3	$[\begin{array}{c} 1\times 1\\ 3\times 3\\ 1\times 1 \end{array}\begin{array}{c} 64\\ 64\\ 256 \end{array}]\times 3$
	1 × 1	Average pool, 3-d fc, softmax

**Table 4:** ResNet-50 hyperparameter settings.

Parameters	Value
batch	8
Learning rate	0.001
Dropout	0.5

**Table 5:** ResNet-50 intensity level classification.

Heat Island Zoning	Level	Heat Island Intensity (HII) Range
Low-intensity heat island area	1	HII ≤ 1 °C
Sub-low-intensity heat island area	2	1 °C < HII ≤ 2 °C
Moderate-intensity heat island area	3	2 °C < HII ≤ 3 °C
Sub-high-intensity heat island area	4	3 °C < HII ≤ 4 °C
High-intensity heat island area	5	HII > 4 °C

**Table 6:** GBA heat island area change of each intensity (km^2^) from 2000~2020.

HII Level/Year	2000	2005	2010	2015	2020	2000–2020
1	40,662.47	35,570.59	33,002.57	27,234.17	29,671.11	−10,991.36
2	7796.40	8175.21	8017.03	9103.64	6810.16	−986.24
3	3746.73	4930.92	4738.77	6450.84	4425.78	679.05
4	1727.53	2938.28	3080.71	3793.95	3426.58	1699.05
5	1566.85	3884.96	6660.90	8917.37	11,166.34	9599.49

## Data Availability

The data for this study are available from the corresponding author upon reasonable request.
